# Stability of Microbiota Facilitated by Host Immune Regulation: Informing Probiotic Strategies to Manage Amphibian Disease

**DOI:** 10.1371/journal.pone.0087101

**Published:** 2014-01-29

**Authors:** Denise Küng, Laurent Bigler, Leyla R. Davis, Brian Gratwicke, Edgardo Griffith, Douglas C. Woodhams

**Affiliations:** 1 Institute of Evolutionary Biology and Environmental Studies, University of Zurich, Zurich, Switzerland; 2 Institute of Organic Chemistry, University of Zurich, Zurich, Switzerland; 3 Center for Species Survival, Conservation and Science, National Zoological Park, Smithsonian Institution, Washington DC, United States of America; 4 El Valle Amphibian Conservation Center, El Valle, República de Panamá; 5 Smithsonian Tropical Research Institute, Balboa, Ancón, Panamá, República de Panamá; Louisiana State University, United States of America

## Abstract

Microbial communities can augment host immune responses and probiotic therapies are under development to prevent or treat diseases of humans, crops, livestock, and wildlife including an emerging fungal disease of amphibians, chytridiomycosis. However, little is known about the stability of host-associated microbiota, or how the microbiota is structured by innate immune factors including antimicrobial peptides (AMPs) abundant in the skin secretions of many amphibians. Thus, conservation medicine including therapies targeting the skin will benefit from investigations of amphibian microbial ecology that provide a model for vertebrate host-symbiont interactions on mucosal surfaces. Here, we tested whether the cutaneous microbiota of Panamanian rocket frogs, *Colostethus panamansis*, was resistant to colonization or altered by treatment. Under semi-natural outdoor mesocosm conditions in Panama, we exposed frogs to one of three treatments including: (1) probiotic - the potentially beneficial bacterium *Lysinibacillus fusiformis*, (2) transplant – skin washes from the chytridiomycosis-resistant glass frog *Espadarana prosoblepon*, and (3) control – sterile water. Microbial assemblages were analyzed by a culture-independent T-RFLP analysis. We found that skin microbiota of *C. panamansis* was resistant to colonization and did not differ among treatments, but shifted through time in the mesocosms. We describe regulation of host AMPs that may function to maintain microbial community stability. Colonization resistance was metabolically costly and microbe-treated frogs lost 7–12% of body mass. The discovery of strong colonization resistance of skin microbiota suggests a well-regulated, rather than dynamic, host-symbiont relationship, and suggests that probiotic therapies aiming to enhance host immunity may require an approach that circumvents host mechanisms maintaining equilibrium in microbial communities.

## Introduction

Environmental changes leading to disruption of the host microbiota, or dysbiosis, can lead to disease emergence [1], [2], [3]. Recent theory has focused on disturbance responses for microbial communities and factors important for community stability [4], [5]. Besides permanent change to an altered stable state, there are four potential alternative responses to disturbance: (1) Resistance – the microbial community does not change, (2) Resilience – the microbial community is changed initially but then returns to its original composition, (3) Functional redundancy – the microbial community changes while maintaining the function of the original composition, and (4) Restoration – the microbial community recovers from a previously degraded state. Most microbial communities in the environment, for example soil communities, tend to be sensitive and not resistant to disturbance [5], [6]. However, few studies have examined disturbances of host-associated microbial communities, and active host regulation of microbiota may produce greater stability than found in environmental microbial communities. Indeed, a number of host processes have been described directing the restoration or recovery of microbiota following disturbance [7].

Intentional disturbances such as antibiotic treatments intended to prevent or manage disease have the unintended effect of disrupting beneficial microbiota [8]. Transfer of microbiota from healthy to diseased hosts (e.g., fecal transplant) has proven more effective than antibiotics against *Clostridium difficile*, and has renewed interest in this type of therapy [9], [10]. Mutualistic microbial communities are linked with the health of organisms across a broad taxonomic range [11], [12]. In amphibian populations threatened by emerging disease, the microbial community response to potential probiotic treatments is critical for effective conservation management [13].

A diverse microbiota has been found on amphibian skin [14]–[20]. These cutaneous microbial communities extend host immune function and are important in the prevention or outcome of diseases such as chytridiomycosis, which is caused by the pathogenic chytrid fungus *Batrachochytrium dendrobatidis* (*Bd*) in amphibians [21], [22]. The fungus is globally distributed on hundreds of amphibian species [23]. While the impacts of infection differ among species and depend on environmental context [24], [25], severe outbreaks can lead to extinctions or collapse of regional amphibian faunas [26], [27] and ecosystem alterations [28]. In Panama, populations of the rocket frog, *Colostethus panamansis* Dunn 1933, declined dramatically [27] and Koch’s postulates were fulfilled for *Bd* as the causative agent of chytridiomycosis [29]. While this species was extremely sensitive to the disease, others, such as the glass frog *Espadarana prosoblepon* Boettger 1892, were able to persist in smaller populations [27], [30].

Growth of the fungus *Bd* is often inhibited by amphibian skin microbiota [17]–[19], [31], [32], and probiotic application of antifungal bacteria is a promising tool for disease mitigation [24], [33]. While sometimes effective, probiotic therapy has met with mixed results [34]–[36]. Probiotic screening protocols and advances in application method are under development [13]. Overcoming hurdles in effective probiotic therapy will involve a better understanding of the microbial ecology of amphibian skin, including the microbial responses to disturbance, and the host responses that maintain a functional microbiota.

Amphibian immune defenses are quite sophisticated and include most components present in mammals [37]. One immune component of particular relevance to skin infections includes release of bioactive compounds into the mucosal layer. Antimicrobial peptides (AMPs) are synthesized in dermal granular glands of many species [38]–[42]. AMPs are stored in the granular glands and released to the skin surface when the animal is alarmed or injured, and small amounts are constitutively expressed [43]. The response is thought to be a non-specific and fast-acting innate defense, although AMP responses can be closely linked with adaptive immunity [44]. Amphibian skin peptides can directly inhibit amphibian pathogens such as *Bd*, *Basidiobolus ranarum* or ranaviruses in vitro [45]–[47]. Interactions between AMPs, microbiota, and environmental conditions may be important for maintaining a functionally stable microbiota and is an ongoing area of study [48]–[50].

Here, we examine AMP responses to three microbial treatments of a threatened Central American amphibian. Frogs were treated with a probiotic skin bacterium, treated with skin-wash transplants from a disease-resistant species, or treated with sterile water as controls. Resistance of the microbiota to colonization by exogenous microbes would indicate that host mechanisms are maintaining homeostasis. This knowledge will aid strategies to enhance immune function and mitigate disease.

## Materials and Methods

### Study Species and Sites

In January 2011, we collected rocket frogs, *C. panamansis* (n = 44), from a stream at the Sierra Llorona Lodge near Colón, Panama. Glass frogs, *E. prosoblepon* (*n* = 17), were collected from Omar Torrijos National Park, Coclé Province, Panama. Frogs were transported to the El Valle Amphibian Conservation Centre (EVACC) at El Níspero Zoological Park, El Valle, Panama. Collecting permits were provided by the Autoridad Nacional del Ambiente (ANAM), and all experimental procedures were approved by the Smithsonian Tropical Research Institute (STRI) Institutional Animal Care and Use Commission. After treatments, frogs were monitored daily to record microhabitat use, behavior, and animal welfare. After the experiment, all animals were retained in captivity at EVACC and not released, according to ANAM specifications.

### Animal Care

All *C. panamansis* were housed individually in 60 L plastic mesocosms situated on a shaded lawn at EVACC. The tubs were filled with 2 to 3 L of filter-sterilized water and tilted to one side to cover approximately one third of the surface, and a large rock was provided as a hide in the dry portion of the enclosure. Water was exchanged every 8 d and waste was decontaminated with bleach before disposal. Frogs were fed with small domestic crickets (*Acheta domesticus*), fruit flies (*Drosophila hydei*), or a mixture of both every other day. All *E. prosoblepon* were housed indoors in individual 2 L plastic enclosures containing water and large leaves. They were fed with *D. hydei* every other day.

### Experimental Design

Frogs were captured by hand using a fresh pair of gloves for each capture. Before swabbing the frogs, they were rinsed with approximately 20 ml filter sterilized water to remove debris and transient bacteria not associated with the skin [15]. Upon capture, each *C. panamansis* was swabbed twice with a sterile rayon swab (Copan, Brescia, Italy) on the thighs, hands and feet 5 times and 10 times on the ventral surface. Swabs were then immediately stored at −15°C. The first swab was used for the analysis of the microbial skin community as described below, and the second swab was given to Roberto Ibáñez at the Smithsonian Tropical Research Institute in Panama City, Panama to test for *Bd* by qPCR according to Boyle et al. [51]. Rocket frogs were distributed randomly among three treatment groups. Glass frogs, *E. prosoblepon*, were collected on January 11 and 12, and rocket frogs were collected on January 13–15. After determining *Bd* infection status of all individuals, treatments of rocket frogs began January 24 (day 1). A second swab for microbial skin community analysis was obtained on day 48, at which time skin peptides were also sampled, marking the end of the experiment and a biologically relevant time point for assessing changes in skin peptides. Collection of microbes and skin peptides from *E. prosobleopon* occurred as soon as possible after use of frogs in the experiment (day 14).

#### Probiotic treatment

Frogs (n = 15) were exposed to the antifungal bacterium, *Lysinibacillus fusiformis*. In January 2010, *L. fusiformis* was isolated from the skin of a *C. panamansis* from Tortí, Panama and cryopreserved at STRI until use. The isolate was identified by Matthew Becker at Virginia Tech University and the 16S rDNA sequence has been deposited in the EMBL Nucleotide Sequence Database (accession number HE817768). The host frog was not infected with *Bd*, although some other amphibians at the site were *Bd* positive including two *Craugastor crassidigitus* out of 93 sampled amphibians. Some strains of the bacterium can produce tetrodotoxin [52] and have the capacity to inhibit *Bd* growth in the laboratory. Thus, Dendrobatid frogs may form symbioses with this bacterium to increase antimicrobial or anti-predator defenses. Bacteria were incubated for 96 h at room temperature on glucose-casein-KNO_3_ agar plates (containing 0.5 g glucose, 0.3 g casein, 2 g KNO_3_, NaCl and K_2_HPO_4_, 0.05 g MgSO_4_*7H_2_O, 0.03 g CaCO_3_, 0.01 g FeSO_4_*7H_2_O and 20 g agar per L medium). After incubation, *L. fusiformis* was washed from the agar plate with filter-sterilized water and 25 ml of the bacterial solution was poured into each of fifteen 50 ml centrifuge tubes. Rocket frogs were placed individually into the tubes for 1 hr. This treatment was performed once, on day 1 of the experiment.

#### Transplant treatment

Frogs (n = 15) were exposed to skin washes from *E. prosoblepon* (*n* = 16). Glass frogs appear to have an exaptation to resist *Bd* and pre-existing mucosal defenses that protect nests from pathogenic fungi [32]. Glass frogs were given daily washes in 25 ml of filter-sterilized water for 30 min. All skin washes were then mixed together and 25 ml applied to each of the 15 *C. panamansis* for 30 min in 50 ml centrifuge tubes. Starting on day 1 of the experiment, the treatment was repeated once daily for 7 d.

#### Control treatment

Frogs (n = 14) were held as controls with a handling regime matching that of the transplant treatment. Control frogs were given a daily wash in 25 ml filter sterilized water for 30 min each of 7 d starting on day 1 of the experiment. Any affect of stress from handling or daily treatment washes was matched in these controls.

### Data Collection

#### Frog mass

The mass of all *C. panamansis* was measured on day 1, day 28 and day 48 of the experiment. To test for treatment effects on mass we used repeated measures ANOVA. Slope of mass change through time was compared among treatment groups by ANOVA with Tukey HSD pairwise comparisons. All statistical tests were carried out with IBM SPSS Statistics v. 19 (SPSS Inc.) unless otherwise indicated, and non-parametric tests were used when data were not normally distributed and had heterogeneous variances (Levene’s test, P>0.05).

#### Microbiota

Dynamics of bacterial communities were investigated by terminal-restriction fragment length polymorphism (T-RFLP), a consistent and high-resolution culture-independent technique used to monitor microbial community changes over space or time [53]–[56]. DNA was extracted from swabs with the Microbial Ultra Clean DNA Kit (MO BIO) following the protocol of the manufacturer. Bacterial 16S rDNA was then amplified using the primer 27F (PET® labelled) (5′-AGA GTT TGA TCC TGG CTC AG-3′) and 1492R (5′-TAC GGY TAC CTT GTT ACG ACT-3′) (Applied Biosystems). For the 20 µl PCR reaction mixture, the thermocycling conditions were set as follows: 95°C for 5 min followed by 32 cycles of 94°C for 1 min, 52°C for 1.5 min, 72°C for 2 min and a final elongation for 10 min at 72°C. Each reaction contained the following reagents: 2 µl template DNA, 1 µl of each primer (10µM), 1 µl BSA (2 ug/µl), 2 µl dNTPs (2 mM), 4 µl buffer (5x), 1.2 µl MgCL_2_ (25 mM), 1 µl Taq 1∶10 (0.5 u/µl), and 6.8 µl water.

To minimise PCR bias, PCR reactions were run in triplicate and their products combined and checked by electrophoresis in 1% agarose with GelRED staining. In order to eliminate pseudo-terminal fragments [60],10 units of mung bean nuclease and 12 µl of 10x reaction buffer were added to the PCR product to digest single-stranded DNA. The digestion was performed with a total volume of 120 µl at 30°C for 2 h. To stop the reaction, SDS (0.1%) was added to a final concentration of 0.01%. Mung bean digested PCR products were purified with the GenElute PCR Clean-Up Kit (Sigma-Aldrich) according to the protocol provided by the manufacturer until step IV and then eluted with 33 µl of Elution Solution.

Using a spectrophotometer (NanoDrop), the absorption of samples at 260 nm was determined to quantify DNA. Samples were diluted with Milli Q water to a DNA concentration of 50 ng µl^−1^ and aliquots of the samples (200–500 ng) were digested with restriction enzymes. For the 20 µl restriction reaction, 1.5 units of either HaeIII or MspI were used with 2 µl of 10x FastDigest or 10x Tango buffer respectively.

To determine restriction fragment lengths, 2 µl of digested PCR product were run on an ABI 3730 DNA Analyser (Applied Biosystems) equipped with 36 cm capillaries filled with POP7 polymer. For each sample, we used 17.8 µl HiDi-Formamide (Applied Biosystems) and 0.2 µl GeneScan 500 LIZ size standard (Applied Biosystems). T-RF sizes and quantities, measured in fluorescence units (rfu), were determined with GeneMapper v3.7 (Applied Biosystems) using the AFLP option and the Local Southern size calling method. Peak alignment was done automatically by GeneMapper and peak parameters were set to a polynomial degree of 4, window size of 13 and a minimum of width at half maximum (base pairs) of 2. To exclude possible primer dimers or other artifacts, we analyzed peaks in the range of 80–411 bp (HaeIII) and 80–596 bp (MspI) with intensities of ≥150 RFU. Samples with less than 2 peaks were removed from the data set; in total, 6 samples digested with HaeIII and 3 samples digested with MspI were discarded. By calculating the area of each peak as a proportion of the total area, data were standardized in Microsoft Excel and the resultant data set imported into the statistical software package PAST. T-RFLP data were visualized by non-metric multidimensional scaling (nMDS) and hypotheses tested by analysis of similarity (ANOSIM) using Bray-Curtis coefficient similarity matrix, and by non-parametric multivariate analysis of variance (NPMANOVA). As a second method of microbial community analysis, the number of taxa detected in every sample was counted (assuming that each peak represents one single species) and the Simpson and Shannon indices for diversity were calculated in PAST v2.10. These measures of diversity were used to test for differences among treatment groups by ANOVA or to test for changes in microbial diversity through time with paired t-tests.

#### Skin peptides

On day 14 of the experiment, all *E. prosoblepon* were swabbed for microbial community analysis, and afterward, skin peptides were collected. Frogs were given a dorsal subcutaneous injection of 40 nmol norepinephrine (bitartrate salt; Sigma) per g body weight (gbw) to elicit granular gland secretions, then rinsed with 25 ml filter sterilized water and allowed to sit for 15 min. Peptide mixtures were acidified to 1% hydrochloric acid (HCl) to prevent proteolytic degradation of samples. The solution was immediately passed over C-18 Sep-Pak cartridges (Waters Corporation) previously primed with acetonitrile and rinsed, and the Sep-Paks were stored in zip-lock bags with 2 ml of 0.1% HCl. After transport to the University of Zürich, peptides bound to the Sep-Paks were eluted with 70% acetonitrile, 29.9% water, and 0.1% HCl and concentrated to dryness by centrifugation under vacuum, and weighed. The same procedure was used to collect peptides from *C. panamansis* after swabbing them at the end of the experiment for microbial community analysis on day 48.

To test the extracted skin peptides for antimicrobial activity, the growth inhibition of *Bd* zoospores was measured for a subset of samples from each *C. panamansis* treatment group and from *E. prosoblepon.* The dried peptides were dissolved in water and diluted to a concentration of 1000 µg ml^−1^ before addition to a 96-well plate in duplicate. The final peptide concentration was 500 µg ml^−1^ in the wells containing 50 µl peptide and 50 µl *Bd* zoospores in 1% tryptone broth (T broth). Preliminary tests showed that lower concentrations had no significant effects on the growth of zoospores (data not shown). To obtain *Bd*, an RIIA agar plate supplemented with 1% tryptone was inoculated with the panzootic lineage of *Bd* from the UK (generously provided by M. Fisher), grown for 7 d, and flushed with 3 ml of T broth. After a 15 min incubation, the T broth with freshly released and active zoospores at 4.2×10^6^ zoospores ml^−1^ was collected in a sterile reagent reservoir. The 96-well plates were prepared: 100 µl of T broth was added to all outer wells to retain moisture in the plate. Control wells in replicates of 6 contained 50 µl water and 50 µl of *Bd* zoospores heat killed for 15 min at 60°C (negative control) or 50 µl of living *Bd* zoospores (positive growth control). The optical density at 490 nm was measured on days 0 and 7 on a multilabel counter (Victor^3^, Perkin Elmer) and plates were incubated at 18°C, an optimal temperature for *Bd* growth [57]. The percentage of *Bd* growth inhibition for each peptide sample was calculated by comparison to controls. The percentage of *Bd* growth inhibition was then multiplied by the quantity of peptides produced per cm^2^ surface area to calculate the peptide capacity against *Bd*. Peptide capacity is similar to the measure of peptide effectiveness used by Woodhams et al. [58] for small frogs where large quantities of peptides are not available for testing minimal inhibitory concentrations. The skin surface was estimated using the equation: surface area = 9.90*(weight in grams)^0.56^ [59]. We tested for differences among treatments and species in the quantity of peptides recovered, growth inhibition of *Bd* (%), and peptide capacity by ANOVA with Tukey HSD pairwise comparisons.

To test for differences in the composition of skin peptides among the three *C. panamansis* treatment groups, skin peptides were analyzed. Ultra-high performance liquid chromatography electrospray ionization mass spectrometry (UHPLC-ESI-MS) was performed on a Waters Acquity UPLC (Waters, Milford, MA 01757, USA) connected to a Bruker maxis high-resolution quadrupole time-of-flight mass spectrometer (Bruker Daltonics, Bremen, Germany). An Acquity BEH 300 C18 column (Waters, 1.7 µm, 1×100 mm) has been used with a gradient of H_2_O+0.05% TFA (A) and CH_3_CN+0.05% TFA (B) at 0.2 mL/min flow rate (linear gradient from 10 to 50% B within 10 min followed by flushing with 100% B for 4 min). All solvents were purchased in best LC-MS qualities.

The mass spectrometer was operated in the positive electrospray ionization mode at 4′500 V capillary voltage, –500 V endplate offset, with a N_2_ nebulizer pressure of 1.4 bar and dry gas flow of 10.0 L/min at 200°C. MS acquisitions were performed in the mass range from *m/z* 100 to 3′000 at 20′000 resolution (full width half maximum) and 2 scans per second. The MS instrument was optimized for maximum intensities of Bradykinin at *m/z* 530.8. Masses were calibrated with an electrospray calibrant solution (Fluka, Buchs, Switzerland, 20x dilution in CH_3_CN) between *m/z* 118 and 2722.

The relative abundance (area under peak) of 16 peptides was determined by HPLC-MS for a subset of samples from each treatment group and species. The relative intensities of each peptide were ranked to produce a dataset that satisfied Box’s test for homogeneity of covariance matrices. Differences in peptide profiles among treatments were analyzed by multivariate analysis of variance in PAST v2.16. Peptide dry weight was compared with the ranked intensity of the ubiquitous peptide (mass 1064) by linear regression to determine whether dry mass measurements of partially purified skin secretions enriched for hydrophobic peptides could predict the relative abundance of peptide peaks detected by HPLC. To test for a potential immune-energetic trade-off, the quantity of peptides recovered was tested for correlation with change in frog mass. Peptide composition was also tested for affect on frog mass by multiple linear regressions, overall and separately for each treatment.

## Results

### Survival, Bd Infection, and Body Mass

**Figure 1:**
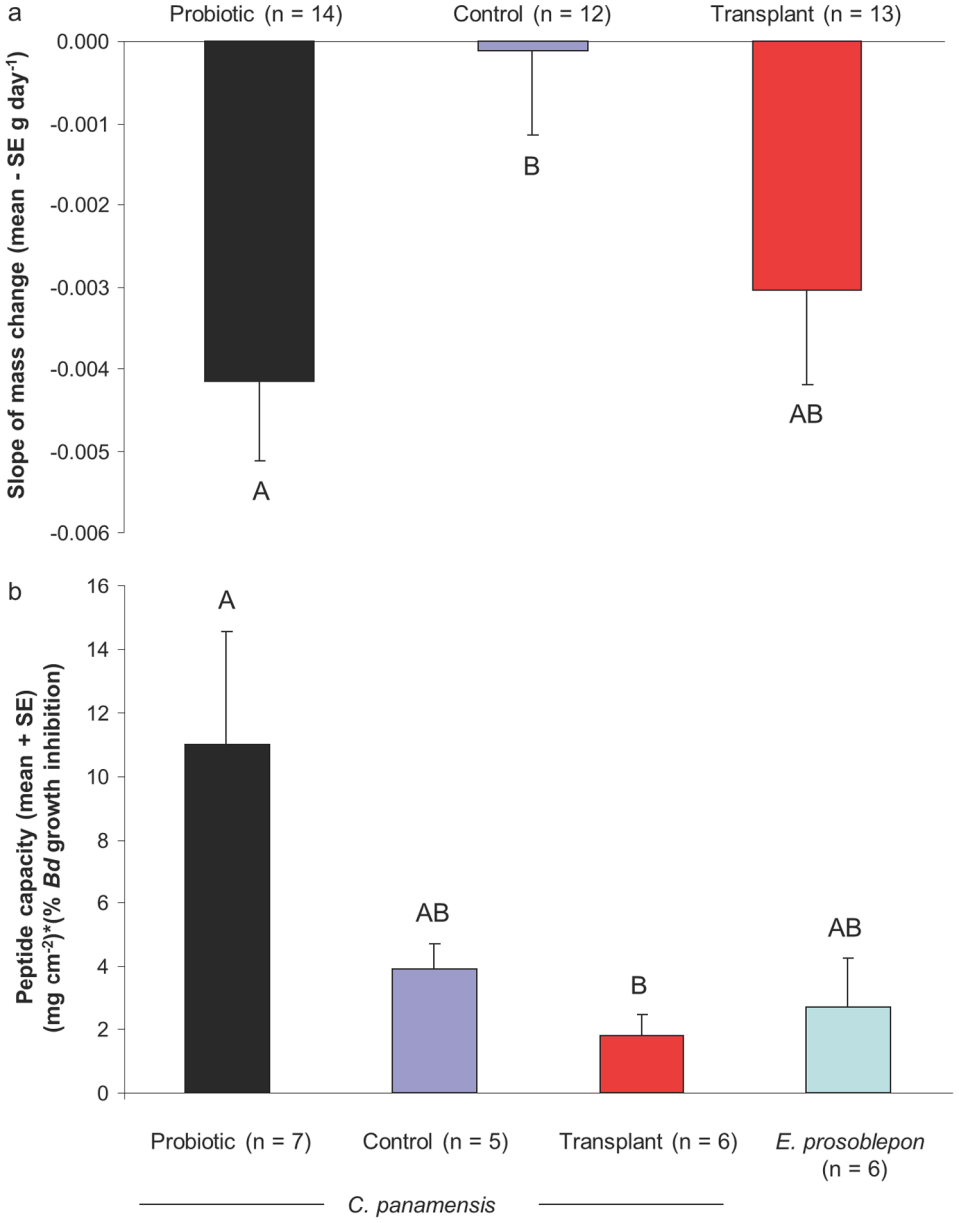
Body mass change and capacity of skin defense peptides against *Batrachochytrium dendrobatidis* (*Bd*) of frogs in different treatments. (a) Slope of mass change. (b) Peptide capacity calculated as peptide quantity per surface area multiplied by percent growth inhibition of *Bd* caused by 500 µg ml^−1^ peptide for each treatment or species. Letters above bars indicate homogeneous subsets based on ANOVA with Tukey HSD.

The survival rates of *C. panamansis* (N = 44) in the three different treatments were as follows: 86.7% for the probiotic treament, 85.7% for the control treatment, and 78.6% for the skin wash transplant treatment. Five frogs, randomly divided among treatments, were found to be infected with *Bd* but none of these frogs died during the experimental period. Mass loss of infected frogs was not significantly different than mass loss of uninfected frogs (Independent t-test, t_37_ = −0.075, P = 0.940). At the start of the experiment, mass of frogs did not differ significantly among treatments (mean +/− SD = 1.52+/−0.28 g, ANOVA, F_2,36_ = 0.667, P = 0.519). Treatment groups differed significantly in the change of body mass through time (Repeated measures ANOVA, Greenhouse-Geisser F_3.3,56.6_ = 3.168, P = 0.027). Mass loss was greatest in frogs treated with *L. fusiformis* (slope of mass loss, ANOVA, F_2,36_ = 3.850, P = 0.031; Fig. 1a). Mass loss was on average 11.9% for probiotic treated frogs, 0.1% for control frogs, and 7.3% for skin-wash transplant treated frogs.

### Microbiota Composition

**Figure 2:**
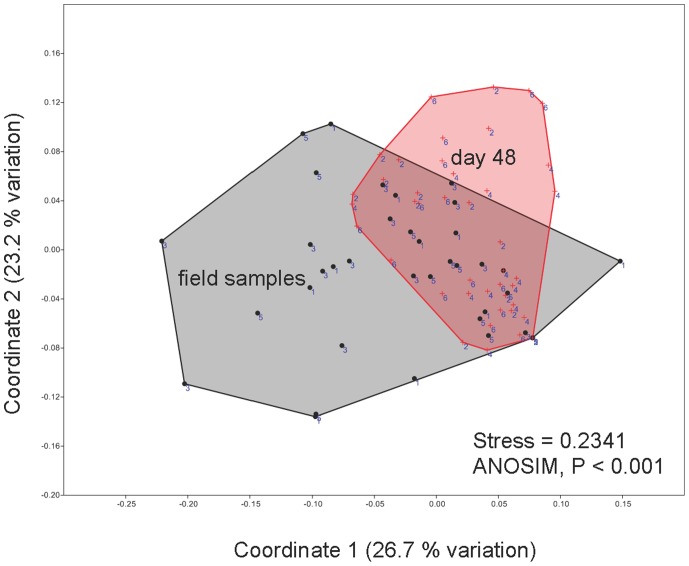
Skin microbial communities of *Colostethus panamansis*. Communities before (circles, field samples) and after (crosses, day 48) treatments visualized by non-metric multidimensional scaling (nMDS) of T-RFLP analysis using enzymes HaeIII and MspI. Treatments are numbered to indicate probiotic bacterium *Lysinibacillus fusiformis* (1,2), control (3,4), and skin-wash transplant from the disease-resistant glass frog *Espadarana prosoblepon* (5,6). Microbial communities were not significantly different among treatments within each time-point represented by convex hulls. Distance between objects on the plot represents relative dissimilarity (axes are in arbitrary units). Stress <0.1 indicates strong community differences and stress >0.2 indicates that differences should be interpreted with caution. Statistical analyses are presented in Table 1.

We did not detect significant differences in the composition of microbiota among the three different groups either before or after treatment (Table 1). The microbiota of all treatment groups changed over time (nMDS, Fig. 2; ANOSIM and NPMANOVA, Table 1), but the stress values of the nMDS plots were high and R values of the ANOSIM low, indicating that the changes were very small. Hence, the microbiota did not differ significantly among treatments at the beginning of the experiment, microbiota shifted to a small degree through time in the mesocosms, and microbiota did not significantly differ among treatments at the end of the experiment. The microbiota found on *E. prosoblepon* was significantly different to that found on *C. panamansis* (treatment groups combined) at all times (NPMANOVA, before: F = 4.814, P = 0.0001; after: F = 3.529, P = 0.0001).

**Table 1:** Statistical analysis of treatment differences in microbial communities described by T-RFLP using either Hae3 or Msp1 enzymes.

		ANOSIM				NPMANOVA			
		Msp1		Hae3		Msp1		Hae3	
		R	p	R	p	F	p	F	p
*before treatment*									
P vs. C	−0.0980		0.9910	−0.0660	0.9170	0.2900	0.9980	0.5100	0.9720
P vs. T	−0.0360		0.7450	−0.0470	0.8270	0.5700	0.8960	0.6000	0.8750
C vs. T	−0.0560		0.9240	−0.0220	0.6420	0.6200	0.8820	0.7900	0.6920
*after treatment*									
P vs. C	−0.0300		0.7490	0.0350	0.1930	0.9600	0.4900	1.2700	0.2040
P vs. T	0.0000		0.4230	−0.0230	0.6220	0.9900	0.4350	0.7800	0.6370
C vs. T	0.0080		0.3600	0.0030	0.3880	1.2200	0.2230	1.0900	0.3240
*before, after*									
Treaments combined	**0.1860**		**0.0001**	**0.1420**	**0.0001**	**5.6830**	**0.0001**	**3.9450**	**0.0001**

Analysis of similarity (ANOSIM) and non-parametric multivariate analysis of variance (NPMANOVA) results are shown of the three *C. panamansis* treatments: P = probiotic treatment, C = control treatment, T = transplant treatment. Significant values identified by ANOSIM and NPMANOVA are indicated in bold.

For terminal restriction fragments (TRFs) hydrolysed with HaeIII, the number of detected taxa varied between 3 and 20 before and 2 and 14 after treatment. For MspI between 2 and 18 taxa were found both before and after treatment. There was no change in the Shannon index of diversity, and similarly the Simpson index, detected for either enzyme over time (paired t-test for combined treatments; HaeIII: t = −1.307, P = 0.200; MspI: t = 1.431, P = 0. 1617) nor among treatment groups.

Using pure culture standards and T-RFs obtained from cleavages of the 16S rDNA by several enzymes, T-RFLP can be used identify species within microbial community profiles [55]. The bacterium *L. fusiformis* was initially detected on one frog that died during the experiment (fragment lengths: 148 for Msp1 and 235 for HaeIII) and was not found on any frogs at the end of the experiment including those treated with live cultures of *L. fusiformis*.

### Skin Peptides

The quantity of peptides measured at the end of the experiment differed significantly among the *C. panamansis* treatment groups (Kruskal-Wallis test, P = 0.025). Frogs treated by skin wash produced significantly less peptide per surface area than frogs treated with *L. fusiformis* (Mann-Whitney U test, P = 0.015), and control frogs were intermediate. Peptide recovery was highest in frogs treated with the probiotic, and frogs in this group also lost the most body mass during the experiment; however, there was not a significant overall correlation between peptide production and change in mass (Pearson correlation, r = −0.175, P = 0.308). Nor was there a significant affect of peptide profile on slope of mass when analyzed by multiple linear regressions overall or separately for each treatment (P’s >0.05).

*Bd* growth inhibition caused by 500 µg ml^−1^ peptides also differed significantly among *C. panamansis* treatments (ANOVA, F_3,23_ = 34.492, P<0.0001) and was greatest in frogs treated with skin washes from *E. prosoblepon*. The capacity of peptide defenses against *Bd* differed significantly among treatment groups and species (Fig. 1b). Frogs treated with *L. fusiformis* had at least twice the peptide capacity to inhibit *Bd* than frogs in any other group (Fig. 1b).

**Figure 3:**
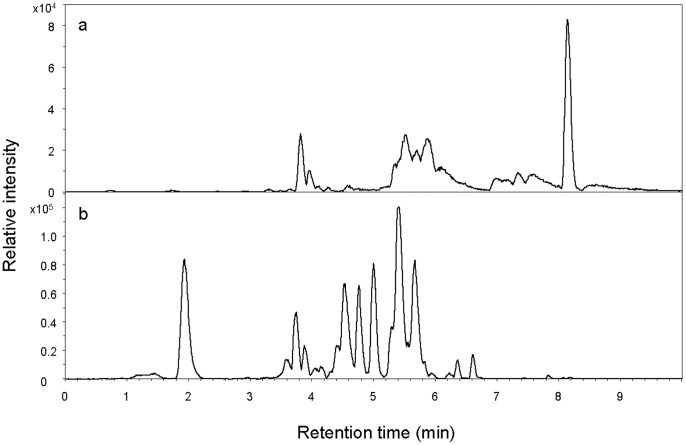
Representative chromatograms of skin defense peptides examined by HPLC-MS. (a) *Colostethus panamansis.* (b) *Espadarana prosoblepon*. Values of molecular weight and mean area for the detected peptides are reported in Table 2.

Rocket frogs, *C. panamansis*, expressed between 1 and 16 skin peptides (mean = 4.8) detected by HPLC (Table 2, Fig. 3). Common skin peptides included molecular weight 1005.6, 1064.0, and 3306.6. Glass frogs, *E. prosoblepon*, produced a different set of skin defense peptides (Table 2, Fig. 3). Primary structures have not been described. Skin peptide profiles were not significantly different among treatments of *C. panamansis* (MANOVA, Wilks’ Lambda, F_18,50_ = 0.756, P = 0.738). Similar results were obtained with non-parametric tests of untransformed data. Overlapping peptide profiles suggest that peptide quantity, rather than profile, was primarily affected by treatment, with the exception of one peptide.

**Table 2:** Retention time (Rt), molecular weight (MW), prevalence, and mean relative area of each peptide based on HPLC-MS chromatograms.

Rt (min)	MW	Prevalence (%)	Mean relative area
*Colostethus panamansis* (N = 36)			
3.6	1041.5	61	0.14
4.45	1064.0	100	1.00
4.5	3306.6	72	0.35
4.8	1936.2	25	0.12
5.0	1512.0	42	0.28
5.3	2988.6	3	0.03
5.3	3001.5	3	0.02
5.7	2986.6	6	0.20
7.0	2974.5	17	0.65
6.8	2957.7	17	0.57
7.1	2972.6	17	0.93
7.0	2290.4	11	1.07
7.3	2231.4	6	0.63
7.6	2315.4	6	0.94
8.1	1005.6	94	1.66
9.7	1790.0	6	0.13
*Espadarana prosoblepon* (N = 16)			
1.45	1746.7	81	0.02
2.2	1585.8	94	0.31
3.7	2650.2	100	0.72
4.4	2698.2	100	1.14
4.55	2634.2	100	0.74
4.6	2652.2	100	0.37
4.75	1004.6	100	0.39
5.05	1004.6	100	0.51
5.45	2681.2	100	1.00
5.69	2665.1	38	0.06

Area is relative to a consistently observed peak, MW 1064.0 for *C. panamansis* and MW 2681.2 for *E. prosoblepon*.

**Figure 4:**
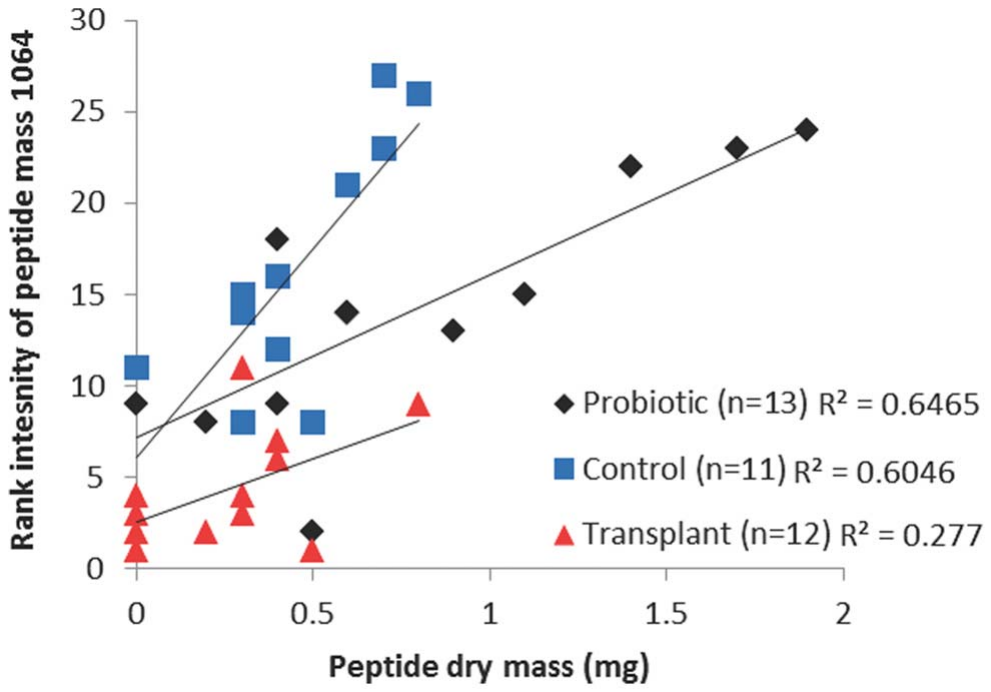
Peptide dry mass predicts a significant proportion of variation in peptide intensity determined by LC-MS. Overall, there was a significant correlation between peptide dry mass and rank abundance of peptide mass 1064. This relationship differed among treatments indicating a change in the relative abundance of the peptide components of skin secretions depending on microbial treatment. Transplant treated frogs had the highest relative abundance of peptide mass 1064 (lowest rank), and probiotic treated frogs the lowest relative abundance with controls intermediate. Probiotic treated frogs had significantly higher total quantity of peptides than transplant treated frogs (see text).

Peptide of mass 1064 was present in all *C. panamansis* samples, and was the only peptide that differed in relative abundance among treatment groups (ANOVA, F_2,33_ = 3.611, P = 0.038). This peptide was most abundant in transplant treated frogs, least abundant in probiotic treated frogs, and intermediate in controls. There was a significant correlation between total peptide dry mass and rank intensity of peptide mass 1064 (Fig. 4; overall Pearson correlation, r = 0.672, N = 36, P<0.001, R^2^ = 0.4522). Treatment accounted for 26.4% of the total variance in rank peptide intensity controlling for the effect of peptide quantity (ANCOVA, F_2,32_ = 17.124, P<0.001, ω^2^ = 0.264). Thus, higher total peptide quantity was associated with lower relative abundance of peptide mass 1064 in a treatment-specific pattern (Fig. 4).

## Discussion

### Stability of Skin Microbiota

Probiotic therapy is a promising disease mitigation strategy currently under development as many amphibian populations decline worldwide [13], [24], [33]. Applications of probiotics may be considered a managed pulse disturbance of the microbial community, but the response to disturbance in terms of stability of host-associated microbiota has not previously been tested. We found that an amphibian species threatened by chytridiomycosis in Panama had a remarkably stable skin microbiota that was resistant to alteration by experimental treatments with skin washes from a co-occurring disease-resistant species, and with the potential probiotic bacterium *L. fusiformis*. Although *L. fusiformis* is a naturally occurring symbiont of *C. panamansis*, and may be responsible for defensive tetrodotoxin compounds found in the skin of Dendrobatids [52], [61], the bacterium did not establish. We did not detect tetrodotoxin production from the bacterium grown in isolation (K. Minbiole, unpublished data). Mechanisms maintaining bacterial communities on amphibian skin have not been previously described. Skin defense peptides extracted from the skin of *C. panamansis* inhibited the growth of the pathogenic fungus *Batrachochytrium dendrobatidis* and contributed to host mechanisms maintaining the microbial composition of *C. panamansis* by limiting *L. fusiformis* and exogenous microbes from *E. prosoblepon* skin washes. Application of *L. fusiformis* led to increased peptide capacity against *Bd* in *C. panamansis*.

Although the probiotic bacterium originally isolated from *C. panamansis* did not establish under our experimental conditions, the composition of the skin microbiota of *C. panamansis* changed over the course of the 48 d experiment in all treatments. Thus, the skin-associated microbial community was resistant to experimental disturbance but showed a gradual shift through time, and was perhaps more influenced by environmental conditions than exogenous microbial exposure. The temperature in the mesocosms at El Valle over the course of the experiment (mean 22.8°C) was probably a little lower than that of the lowland rainforest at Sierra Llorona lodge were the frogs were captured. This factor could also have initiated a shift in the microbial communities on the frog skin. The degree to which amphibians depend on their environment or contact with conspecifics to maintain their microbiota long-term is unknown, but other studies have shown slight changes in microbial diversity through time in captivity [36], [62]. Here, regulation of microbiota by host immune factors [63] is supported.

How amphibians acquire the microbiota on their skin remains unclear. Plausible routes of transmission include contact with conspecifics (horizontal transmission), habitat (environmental transmission), or parents (vertical transmission [32]). Colonization of *L. fusiformis* on the skin of adult *C. panamansis* after contact with high concentrations of bacteria was not successful, and there are several potential explanations. (1) Colonization may begin at early developmental stages when the microbiota reaches a stable equilibrium that then resists disturbance [64], [65]. (2) Competition for resources such as nutrients or space could have prevented establishment of a new member of the skin microbiota [66], [67]. (3) Resident microbiota may have prevented the invasion of *L. fusiformis* by the production of antibiotic metabolites or bacteriocins [68], [69]. (4) Host immune factors in the skin including AMPs [37] may have been induced and excluded *L. fusiformis*.

Antimicrobial defense peptides extracted from the skin of *C. panamansis* differed significantly among treatments in quantity and in relative abundance of peptide mass 1064. This peptide will be targeted in future studies for primary structure determination and for testing of antifungal function. *C. panamansis* exposed to potentially beneficial bacteria and other host factors in the mucus washed from the skin of *E. prosoblepon* did not increase overall peptide quantity, but did show an increase in the relative abundance of peptide mass 1064 and a corresponding increase in *Bd* inhibition at a standardized concentration of 500 µg ml^−1^ peptide. *C. panamansis* exposed to cultured *L. fusiformis* produced greater quantities of AMPs than frogs in the other two treatment groups, leading to greater defense capacity against *Bd* (Fig. 1), and suggesting a generalized induced immune response. Similarly, Schadich et al. [70] described increased peptide production in the frog *Litoria raniformis* induced by exposure to the pathogenic bacterium *Klebsiella pneumoniae*. In this study, induction of skin defense peptides likely contributed to the elimination of *L. fusiformis*, and the inability of the probiotic to establish within the host skin microbial community. At the same time, frogs in the *L. fusiformis* treatment lost significantly more weight than frogs in other treatments, indicating a potential cost to immune activation [71]. Certainly, other host responses in addition to skin peptides may have occurred simultaneously, contributing to the observed treatment effect on mass loss.

### Susceptibility to Chytridiomycosis

Soon after the arrival of *Bd* at Omar Torrijos National Park in 2004, *C. panamansis* populations declined critically [27], [29], whereas the frogs sampled near the Sierra Llorona lodge appeared to be coexisting with *Bd*, with a prevalence of 11.4% (95% binomial confidence interval: 3.8–24.6%). That *C. panamansis* are able to persist in an area with *Bd* may be due to infection tolerance [72] or related to habitat characteristics. Temperatures in the lowland rainforest near Sierra Llorona lodge are typically higher than in the cloud forest habitat at Omar Torrijos National Park. Environmental factors such as temperature can also influence the synthesis and expression of skin defense peptides in amphibians [37], [73]. While none of the frogs in this study showed clinical signs of chytridiomycosis, infection status may be an important driver of immune function, or a response to immune function including AMPs and microbiota, and thus an important target for future investigation. In particular, does microbial therapy have the same effect as a treatment of infection as it does as a prophylactic treatment?

Based on samples taken before *Bd* emergence at Omar Torrijos National Park [58], *E. prosoblepon* skin defense peptides were expected to be more effective against *Bd* growth than *C. panamansis* peptides. Thus, similar or greater *Bd* growth inhibition caused by skin peptides from all three treatment-groups of *C. panamansis* compared to *E. prosoblepon* peptides in this study was unexpected. In contrast to *C. panamansis*, *E. prosoblepon* has been able to survive for more than 8 yr at Omar Torrijos National Park, and 16 yr at Fortuna in the presence of *Bd* [27], [30]. We found higher values of peptide effectiveness against *Bd* than previously reported for *C. panamansis*, and this might be explained by population origin of the frogs. Glass frogs, *E. prosoblepon*, were sampled from the same upland site as in the previous study, while *C. panamansis* were extirpated from the upland site and for this study frogs were captured from a lowland rainforest habitat. AMPs from these frogs may have been up-regulated by exposure to *Bd* or microbiota, or AMP defenses may differ among populations or habitats [74]. Stressors may also differ among sites, and long-term upregulation of stress hormones including glucocorticoids can suppress immunity [75] including AMP skin defenses in amphibians [76]. Besides the invasion of *Bd* at Omar Torrijos National Park, stressors have not been reported [29].

### Considerations for Probiotic Therapy

Promoting and sustaining human health through strategies that manipulate microbial communities is a long-term goal of the Human Microbiome Project [77]. Thus, amphibians and other model vertebrate systems are important for examining host-microbiota interactions to gain a mechanistic understanding of microbial community assembly and maintenance [62]. Probiotic disease mitigation is also high on the list of conservation options available for threatened amphibians [13], [24].

An intuitive strategy of reducing the biomass or diversity of resident skin microbiota may aid in the establishment of new bacteria by minimizing community interactions. However, antibiotic pre-treatment interfered with intestinal microbial community establishment in rats [78]. Becker et al. [35] first washed golden frogs, *Atelopus zeteki*, in a 1.5% solution of hydrogen peroxide to reduce microbiota before probiotic treatment. However, the bacterium *Janthinobacterium lividum* did not establish on the skin of the frogs. Pathogens can become established in hosts treated with antibiotics by exploiting the reduced competitiveness of the disturbed community [79], and intestinal disease has been linked to the outgrowth or loss of certain components of the microbiota [80]–[82]. Conversely, beneficial bacteria can also establish in hosts and many examples of successful probiotic use have been reported in aquaculture [83], livestock and poultry production [84], as well as in human medicine [85], [86].

A recommended step for probiotic application is to use small probiotic doses and to wash bacterial cultures in a physiological solution to ensure that hosts are exposed to the living cells only, minimizing exposure to metabolic products of the bacteria including immunomodulatory toxins [34], [35]. Metabolites from unwashed whole cultures may help bacteria in microbial competitive interactions; however, toxins or inordinately large probiotic doses may also elicit host immune responses. It remains unclear whether pre-treatment steps to reduce endogenous microbiota or to wash beneficial bacteria are necessary to introduce an exogenous bacterium into an existing microbial community, but this is a critical consideration for use of probiotics in disease management. The bacterium *J. lividum*, used by Harris et al. [34] on *Rana muscosa* was likely already present on many of the frogs and represents a bio-augmentation experiment. Thus, altering relative population sizes and community function within an established microbiome may be more feasible than altering community membership.

The composition of microbiota can affect host immune responses and influence disease outcome. For individuals with functional skin microbiota and immune defense, colonization resistance can be beneficial, for example in times of environmental change. On the other hand, a resistant or resilient microbiota is not desirable for enhancing host disease resistance through probiotic therapy. Probiotic therapies aim to alter the microbial community to a new stable state that is more protective than the previous state [7]. Establishment of novel microbiota may require methods to circumvent host mechanisms maintaining the microbiota. In the case of *C. panamansis* from Panamanian lowlands, the combination of microbiota that are resistant to colonization, and AMPs effective at inhibiting *Bd* growth, may favor infection tolerance and population persistence. The continuing development of probiotic strategies offers hope for populations threatened by infectious disease.
